# Effects of a staged integral art-based cognitive intervention (SIACI) program in older adults with cognitive impairments: protocol for a randomized controlled trial

**DOI:** 10.1186/s12877-022-02961-4

**Published:** 2022-04-07

**Authors:** Yuan-jiao Yan, Ming-ping Ma, Wen-chao Cai, Chen-shan Huang, Rong Lin, Yu-fei Chen, Hong Li

**Affiliations:** 1 Research Center for Nursing Theory and Practice, Fujian Provincial Hospital, Shengli Clinical Medical College of Fujian Medical University, No.134 Dongjie Street, Gulou district, Fuzhou, 350001 Fujian Province China; 2 Department of Nursing, Fujian Provincial Hospital, Shengli Clinical Medical College of Fujian Medical University, No.134 Dongjie Street, Gulou district, Fuzhou, 350001 Fujian Province China; 3 The School of Nursing, Fujian Medical University, No.88 Jiaotong Road, Fuzhou, 350004 Fujian Province China; 4 Department of Radiology, Fujian Provincial Hospital, Shengli Clinical Medical College of Fujian Medical University, No. 134 Dongjie Street, Gulou District, Fuzhou, 350001 Fujian Province China; 5 Department of Chinese Language and Literature, Xiamen University, No. 422 Siming South Road, Xiamen, 361005 Fujian Province China

**Keywords:** Art, Non-pharmacological intervention, Older adults, Randomised controlled trial

## Abstract

**Background:**

Given the aging population worldwide and the COVID-19 pandemic, which has been found to be associated with a deterioration in Alzheimer’s disease (AD) symptoms, investigating methods to prevent or delay cognitive decline in preclinical AD and AD itself is important. The trial described in this protocol aims to evaluate the effects of a staged integral art-based cognitive intervention (SIACI) in older adults with CIs (preclinical AD [SCD or MCI] and mild AD), in order to gather evidence on the effects of SIACI on cognition and psychological/psychosocial health gains and determine the mechanisms.

**Methods:**

The planned study is a single-center, parallel-arm, randomized controlled trial with allocation concealment and outcome assessor blinding. A total of 88 participants will be randomized to two groups: (i) an intervention group that receives the 16-week, 24-session SIACI program and (ii) a waitlist control group (which will receive the SIACI program after completing the follow-up assessment). Global cognitive function, specific domains of cognition (memory, language, executive function, and visuospatial skills), and other health-related outcomes (quality of life, anxiety, depression, sleep quality, and physical activity level) will be measured at baseline, immediately after the intervention, and at the 6-month follow-up. Blood biomarkers, event-related potential (ERP)-P300, and magnetic resonance imaging (MRI) data will be collected at baseline and immediately after the intervention to explore the mechanisms of SIACI.

**Discussion:**

The trial will elucidate the immediate and long-term effects of SIACI based on neuropsychological testing and blood biomarkers, and neuroscience involving ERP-P300 and MRI parameters will make it possible to explore the mechanisms of SIACI in older adults with CIs. The results will provide evidence on the effectiveness of an AT-based cognitive intervention, which may delay or even halt cognitive decline in preclinical AD and AD itself.

**Trial registration:**

ChiCTR, ChiCTR2100044959. Registered 03 April 2021.

**Supplementary Information:**

The online version contains supplementary material available at 10.1186/s12877-022-02961-4.

## Background

Dementia and cognitive impairments (CIs) are some of the most significant issues for ageing populations, and they are becoming increasingly hard to ignore. With an ageing population, China has 264 million people aged ≥60 years [1]. Around 10–11 million people aged ≥60 years in China have dementia, and > 60% of patients with dementia have Alzheimer’s disease (AD) [2]. Additionally, there are approximately 31 million individuals in China with mild cognitive impairment (MCI) [2]. In total, China has approximately 50 million patients with CIs, but most of them are not diagnosed or treated. Social isolation or disengagement due to the COVID-19 pandemic has been found to be associated with a deterioration in AD symptoms and increased symptoms of anxiety and depression, and the pandemic is thought to have accelerated cognitive decline in ageing populations [3]. We speculate that the prevalence of CIs will substantially increase as the population continues to age, and this patient population will have a large effect on society. Along with improvements in health consciousness among people in China, there is a growing concern specifically about brain health and AD, which has prompted more individuals with self-reported cognitive decline to seek medical advice [4].

AD, the most common form of dementia, is a slow, but progressive and irreversible, progressive neurodegenerative disorder resulting in a loss of abstract thinking, memory, language, orientation, and ultimately impaired daily functioning that leads to loss of independence [5]. MCI is a syndrome characterized by mild cognitive decline in one or more domains (such as memory, language, attention, visuospatial skills, information processing speed, and executive function), but remain largely intact and independence is preserved [6]. Subjective cognitive decline (SCD) occurs when individuals experience a subjective decrease in cognitive function, but cognitive performance based on neuropsychological testing and daily functioning shows no evidence of objective CIs, and biomarkers of preclinical AD (such as amyloid-β [Aβ] deposition and grey matter volume loss) are not present [4]. MCI and SCD are currently generally considered to represent preclinical AD [7]. Almost 50% of cases of preclinical AD will progress to dementia, and approximately 60% of affected individuals are estimated to progress to AD, highlighting the importance of early intervention for CIs [8, 9]. The emergence of COVID-19 highlights the need for large efforts to develop new technologies and other interventions to improve the prevention, treatment, and management of CIs, as small COVID-19 outbreaks could arise at any time in any population, requiring social isolation.

Notably, in individuals with AD-related CIs (who have impaired memory, language, and reasoning), the frequent concomitant emergence of depression, anxiety, and personality changes may further erode quality of life [10, 11]. For individuals with preclinical AD or AD itself, a targeted, holistic intervention may preserve both cognition and psychological/psychosocial health. More specifically, it may slow the cognitive decline by capitalizing on the existing cognitive reserve to deliver direct skill training and/or training regarding compensatory, affective, and/or social strategies that may increase brain plasticity in CIs, potentially even restoring normal ageing [12, 13]. As there is a substantial body of evidence on the role of cognitive reserve in shaping AD onset and progression, more attention has been directed to non-pharmacological therapeutic interventions. These interventions are designed to improve cognitive engagement among older adults, and they may alleviate cognitive decline and improve psychosocial aspects of AD-related MCI and AD itself without significant adverse effects [14]. In particular, art therapy (AT) has been developed as a strategy to preserve the cognition and psychological/psychosocial health of older adults, decreasing the progression of dementia even in the preclinical stage [15, 16].

AT is a form of psychotherapy that involves using art-making activities to enhance physical and mental health, which can be tailored to meet a range of needs for different individuals [17]. Art-making activities include performance arts (music, drama, and dance), visual arts (painting, collage, knitting, sculpture, and pottery), literary arts (novel-writing, poetry, and other text-related activities), and the huge range of applied arts (art design, architectural design, and industrial art). AT is thought to be effective because of its biological and humanistic mechanisms. Biologically, AT can engage participants in several cognitive processes, such as planning, creativity, verbal expression, decision-making, cognitive control, and abstract thinking, which may provoke plasticity in the neural pathways that mediate creative cognition and perceptuomotor integration [18–20]. As for the humanistic mechanism, AT is related to person-centered theory [21], and it can offer the opportunity for self-expression, social interaction, and emotional relief in a failure-free environment, which promotes psychosocial wellbeing [22–24]. Seifert et al. [25] reported that for individuals with dementia, AT-based interventions improved mental state, concentration, corporeal memory, self-esteem, self-reliance, and physical activity. Mahendran et al. [26] found that older adults with MCI who received AT had better outcomes than those who received usual care, with significant improvements in memory, attention, visuospatial skills, and executive function at 3 months and sustained improvements in memory at 9 months. Additionally, functional magnetic resonance imaging (fMRI) of retired adults who participated in weekly visual art creation indicated that there was improved brain region interactions (particularly between the frontal, posterior, and temporal brain regions), suggesting that art creation could be an important tool to prevent cognitive decline in older adults [20]. Collectively, these studies contribute to the limited literature on the effects of AT on cognition and psychological/psychosocial health in older adults, and they provide evidence that AT induces plasticity in neural pathways that mediate creative cognition and perceptuomotor integration.

Although no curative pharmacotherapy currently exists for AD, a growing number of studies have documented the significant benefits of AT [15]. AT can be adapted to fit the needs of individuals in the earliest stages of SCD to the later stages of severe CIs [27]. Recently, a based on current evidence, no AT type was significantly more effective in dementia patients than the other types [28]. Additionally, the meta-analysis found that studies on a combination of visual art, music, and drama interventions found significant improvements in at least one outcome measure, such as mental stimulation, verbalization, personal control, positive emotional reactions, satisfaction, and self-esteem [28]. This suggests that a combination of therapy types that engage participants in a variety of art activities may be effective for combating cognitive decline. Moreover, for some individuals, ongoing therapy involving only a single type of art may be difficult (due to issues related to the art form, the media used, and/or the individual’s personality characteristics). However, due to the complex mechanisms of AD and the limited research on combination AT, little is known about combination AT.

To these ends, we aim to conduct a methodologically rigorous randomized controlled trial (RCT) to evaluate the effects of a SIACI program on neuropsychological outcomes in older adults with CIs (preclinical AD [SCD or MCI] and mild AD), in order to gather evidence on the effects of SIACI on cognition and psychological/psychosocial health gains and to determine the mechanisms of SIACI. As most related studies have reported improvements in memory, executive function, or other cognitive domains (based on neuropsychological assessment) [16, 29–32], we plan to investigate the changes in these domains between baseline and after the SIACI. In particular, the participants’ functional brain activity during visuospatial working memory and executive tasks will be assessed by magnetic resonance imaging (MRI) in order to determine whether cognitive improvements in SIACI participants are associated with alterations in functional brain activation (which may underlie potential compensatory mechanisms that improve cognitive performance). In addition, effective interventions modify changes that are known to occur in neurodegenerative diseases (like AD), such as changes in neuroprotective growth factors (e.g., brain-derived neurotrophic factor [BDNF]) [33], inflammatory factors (e.g., interleukin [IL]-6 and tumor necrosis factor [TNF]-α) [34, 35], AD-related pathological biomarkers (e.g., Aβ and phosphorylated tau) [36, 37], cognition-related electrophysiological effects (e.g., event-related potential [ERP]-P300) [38], and reduced neurocognitive functions. We hypothesized that the 16-week, 24-session SIACI will effectively improve neurocognitive performance in older adults with CIs, potentially via divergent molecules (e.g., BDNF, IL-6, TNF-α, Aβ-42, Aβ-40, and phosphorylated and total tau) and neural pathways.

## Methods

### Study aim, design, setting, and ethics

**Fig. 1:**
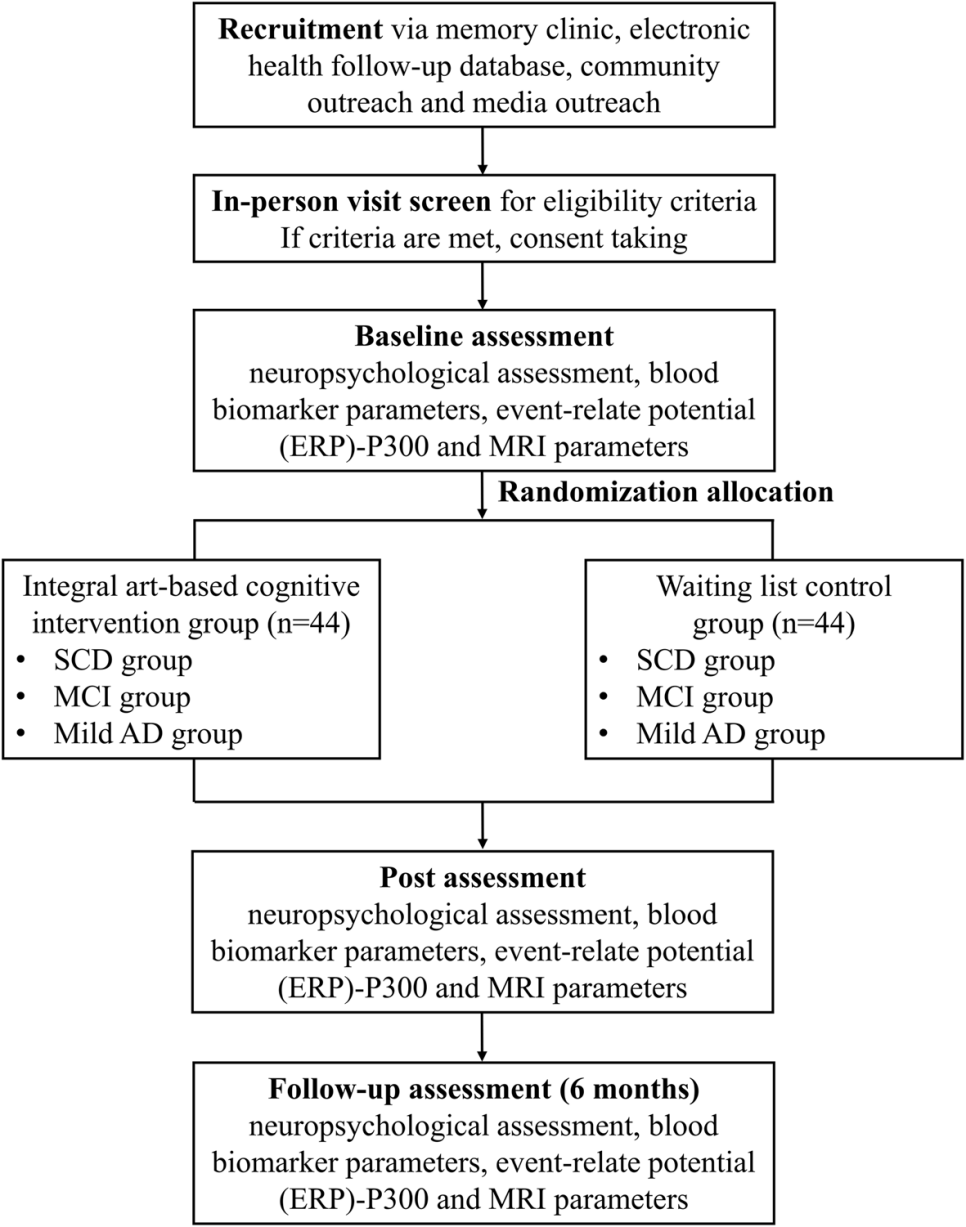
SIACI study flowchart

To evaluate the effectiveness and explore the mechanisms of a SIACI regarding preventing or delaying cognitive decline in older adults, a single-center, parallel-arm RCT will be conducted, with allocation concealment and outcome assessor blinding (Fig. 1).

A total of 88 eligible participants will be randomized to the 16-week, 24-session SIACI group and 16-week waitlist control group. This study will take place at Fujian Provincial Hospital, which is the largest tertiary grade A comprehensive hospital in Fujian Province, China. It is affiliated with the Fujian Provincial Medical Geriatrics Centre, Image Quality Control Centre, and Clinical Laboratory Centre. It is also the largest Public Experiment Service Platform, with highly developed support and laboratory facilities. Data collection for the trial is expected to be completed by late 2023.

As the study involved the older adults with CIs, a few specific ethical procedures were followed and ensured. Only those who signed the consent form would be included in this study, and a guardian as the legal representative was asked to co-sign the consent form. All procedures will be conducted according to the Declaration of Helsinki. The trial has been registered in the Chinese Clinical Trials Registry (ref. ChiCTR2100044959).

### Participants

#### Recruitment and screening

Eighty-eight participants are currently being recruited for the 16-week trial. There are multiple challenges associated with recruiting older adults with CIs into nonpharmacological trials, including stringent medical eligibility criteria, issues ensuring sample representativeness, and the time and effort required of participants that impact motivation to enroll. Thus, to ensure successful and timely enrollment, we are using proactive recruitment strategies, including memory clinic referrals, searches of electronic health follow-up records of the hospital, community outreach (communities, which are near the study hospital, using posters, leaflets and brochures, educational presentations, and a recruiting stations), and media outreach (e.g., local newspapers and online media have published our previous work on the potential benefits of a visual art-based intervention for brain health in MCI patients). If there is restricted site access among the participants during the study because of COVID-19, the time window between baseline and follow-up assessments will be increased to approximately 2–4 weeks.

Potential participants who respond to the recruitment strategies and contact the researchers will be carefully screened for study eligibility at an in-person visit. Participants who meet the inclusion and exclusion criteria will be enrolled, receive information about the trial, and have a discussion with the clinical research coordinator regarding this information. Those who are willing to participate will undergo baseline assessment.

#### Diagnostic criteria

MCI will be diagnosed based on the Peterson diagnostic criteria of MCI [39], as follows: (1) self-reported memory problems, preferably confirmed by someone close to the participant; (2) memory impairment in accordance with age and education (Montreal Cognitive Assessment [MoCA] [40] score of 13–14 for individuals with no formal education, 19–20 for those with 1–6 years of education, and 24–25 for those with ≥7 years of education); (3) intact activities of daily living (ADL) [41, 42] (ADL score < 25 for age ≥ 75 and < 23 for age < 75); and (4) absence of dementia (Mini-Mental State Examination [MMSE] [43] score of 24–30).

SCD will be diagnosed based on the two major features described by the working group of the Subjective Cognitive Decline Initiative (SCD-I) [44], as follows: (1) a self-experienced persistent decline in cognitive capacity (compared to a previously normal cognitive status), which is unrelated to an acute event, and (2) normal performance on standardized cognitive tests used to classify MCI, adjusted for age, sex, and education.

AD will be diagnosed based on the National Institute of Neurological and Communicative Disorders and Stroke-Alzheimer Disease and Related Disorders Association (NINCDS-ADRDA) diagnostic criteria for probable AD [45], and the presence of mild AD will be determined based on an MMSE score of 21–26.

#### Inclusion criteria

1. Diagnosis of SCD, MCI, or mild AD;
2. Age ≥ 60 years;
3. Visual and auditory acuity adequate for neuropsychological testing;
4. Informed consent provided by the participant or a family member.

#### Exclusion criteria

1. Severe aphasia, physical disability, psychiatric disorder, suicide attempt, or other factors that could preclude cognitive examination or completion of the SIACI program;
2. Other neurological or psychiatric conditions that could affect cognition (e.g., Parkinson’s disease, stroke, schizophrenia, severe depression, frontotemporal lobe dementia, Huntington’s disease, brain tumors, metabolic encephalopathy, encephalitis, multiple sclerosis, epilepsy, brain trauma, or normal pressure hydrocephalus);
3. Other systemic diseases likely to impair cognition (e.g., hypothyroidism, Wilson’s disease, folic acid or vitamin B12 deficiency, or viral infection [syphilis or HIV]);
4. Alcohol or drug misuse;
5. Participation in another clinical study at the same time;
6. Acute infectious disease or taking nonsteroidal anti-inflammatory drugs or immunosuppressants in the past month (positive results will exclude participants only from the blood biomarker tests);
7. Metal implant or other MRI contraindications (these will exclude participants only from the MRI examination).

#### Sample size determination

The required sample size was estimated using PASS v11.0 (NCSS, Kaysville, UT, USA) based on a completely random design for comparing the means of two independent samples. To our knowledge, there is no previous study on a comprehensive intervention for CIs among older adults in mainland China, so we estimated the effect size of one of the primary outcomes (general cognitive function) based on our previous study that used creative expression therapy to improve MCI patients’ cognitive function [46] . The study was like the planned trial in some respects, as both are RCTs, one of the primary outcomes is general cognitive function, and one of the primary outcome measurement tools (MoCA) is the same. Our previous study [46] found MoCA scores of 24.68 ± 1.84 and 23.13 ± 1.68 in the intervention and control groups, respectively. A sample size of 35 participants per group was determined to be sufficient to detect an effect with a type 1 error rate of 5% (α = 0.05) and 95% power (β = 0.05). Taking into consideration a 20% attrition rate, a total of 88 participants will be needed, with 44 participants per group.

#### Randomisation, allocation concealment, and blinding

Ensuring allocation concealment, participants will be randomized (after obtaining written informed consent, eligibility screening, and baseline assessment) to the SIACI and waitlist control groups at a 1:1 ratio by a study staff member (who will not be involved in participant recruitment or outcome assessment) using Research Randomizer software (http://www.randomizer.org/). The participants will then be told their group assignment by the intervention staff. Due to the nature of non-pharmacological interventions, only the outcome assessors and data analysts (not the participants or intervention staff) will be blinded to group allocation.

### Intervention group

The SIACI program was developed by the research team in consultation with art therapists, neurology experts, and clinical nursing specialists. To ensure standardized administration of the intervention, it will be delivered under the supervision of qualified art therapists by intervention staff who will have received AT training. The intervention staff will be required to complete an intervention-related competency examination before administering the intervention. In each session, the intervention staff will keep a log of the progress and responses of each participant.

AT, with appropriate modifications, is suitable for all dementia phases [27, 47]. Participants with different degrees of CIs will be assigned to different groups corresponding to their ability. The group size will be based on Liebmann’s [48] suggestion that the appropriate number of participants in group activities is 4–12, but if there are experienced group activity facilitators, this can be increased, as long as all participants can maintain visual contact, communicate verbally, and have the opportunity to share and express themselves.

**Fig. 2:**
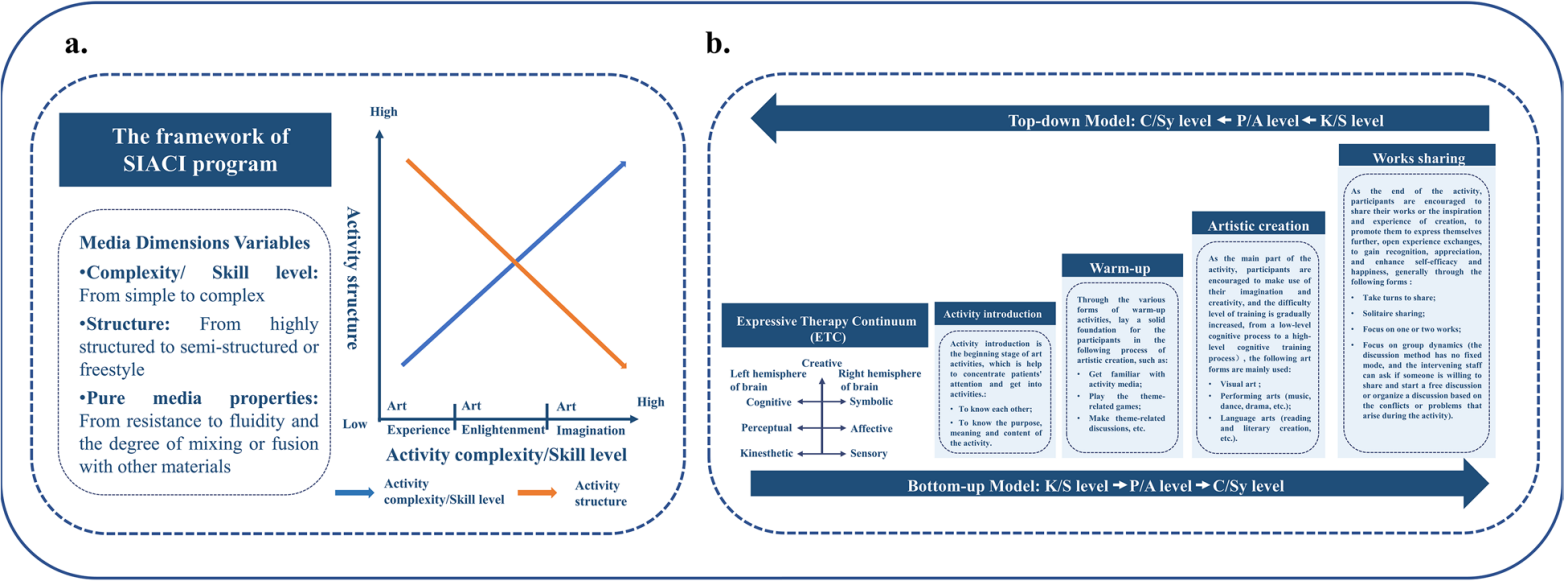
Overview of the framework of SIACI program. **a** The framework of SIACI program. **b** The activity process of SIACI program

The program will be conducted using neurocognitive function training patterns [49], which integrate bottom-up and top-down approaches (Fig. 2). The program will be divided into three modules, i.e., art experience, art enlightenment, and art exploration, which correspond to the three stages of cognition regulation (perception, comprehension, and application). The AT-based cognitive intervention sessions will be based on the theoretical frameworks of media dimension variables (MDV) (Fig. 2A) and expressive therapies continuum (ETC) (Fig. 2B) [50–53]. The activities will include but not be limited to the following diverse artistic expressive forms: performance arts (music, drama, and dance), visual arts (painting, collage, knitting, sculpture, and pottery), literary arts (novel-writing, poetry, other forms of text-related activities), and the huge range of applied arts (art design, architectural design, and industrial art). Each session will follow the principles of individualization, changing the difficulty/cognitive level from simple to complex, changing the activity structure from highly structured to semi-structured or freestyle, and timely adjustments to adapt to and gradually improve cognitive function.

There will be four main activity processes in each session. Firstly, the activity will be introduced to let the participants get to know each other and the purpose, meaning, and content of the activity. Secondly, the participants will be asked to participate in various warm-up activities related to the activity theme, get familiar with the activity materials, and/or have an activity theme-related discussion before the art creation. Thirdly, they will be asked to create anything they want that they think is relevant to the theme for each session. Finally, they will be asked to share their creations and their feelings and perspectives to help them to gain insights and discuss their feelings. The framework of the SIACI program is shown in Fig. 2.

If there is a COVID-19 outbreak in the area or nearby area during the intervention period, and it is therefore necessary to stop the group AT activities, an intervention contingency plan will be carried out. A package containing the activity-related materials will be mailed to each participant’s home, WeChat software (which is the most widely used social media app among older adults in China) will be used as the medium to introduce the activity theme, material descriptions, and other related information, and WeChat groups will be used to communicate and to share the resultant art works. If a participant cannot use a device to access WeChat (such as a smartphone, iPad) or use WeChat itself, they will receive the required information (the activity theme, material descriptions, and other related information) in paper form and, if necessary, they will receive communication and guidance by phone. Their art works will then be shared when the group art activities are resumed in person, to ensure the ongoing implementation of the intervention as far as possible.

### Waitlist control group

Following baseline assessment, participants in the waitlist control group will engage in their usual activities during the 16-week study period. After the study, if the SIACI program leads to significant improvements in cognitive function, these participants will be offered the program as a courtesy for their participation.

### Outcome measures

To explore the effects and mechanisms of SIACI, various measurements will be obtained at baseline, immediately after the intervention, and at the 6-month follow-up. Outcome measures will include neuropsychological assessments, blood biomarkers, ERP-P300, and MRI parameters. All outcome measures and assessment time points are shown in Table 1. The outcomes will be assessed by experienced staff members who will be blinded to group allocation throughout the study.

**Table 1:**
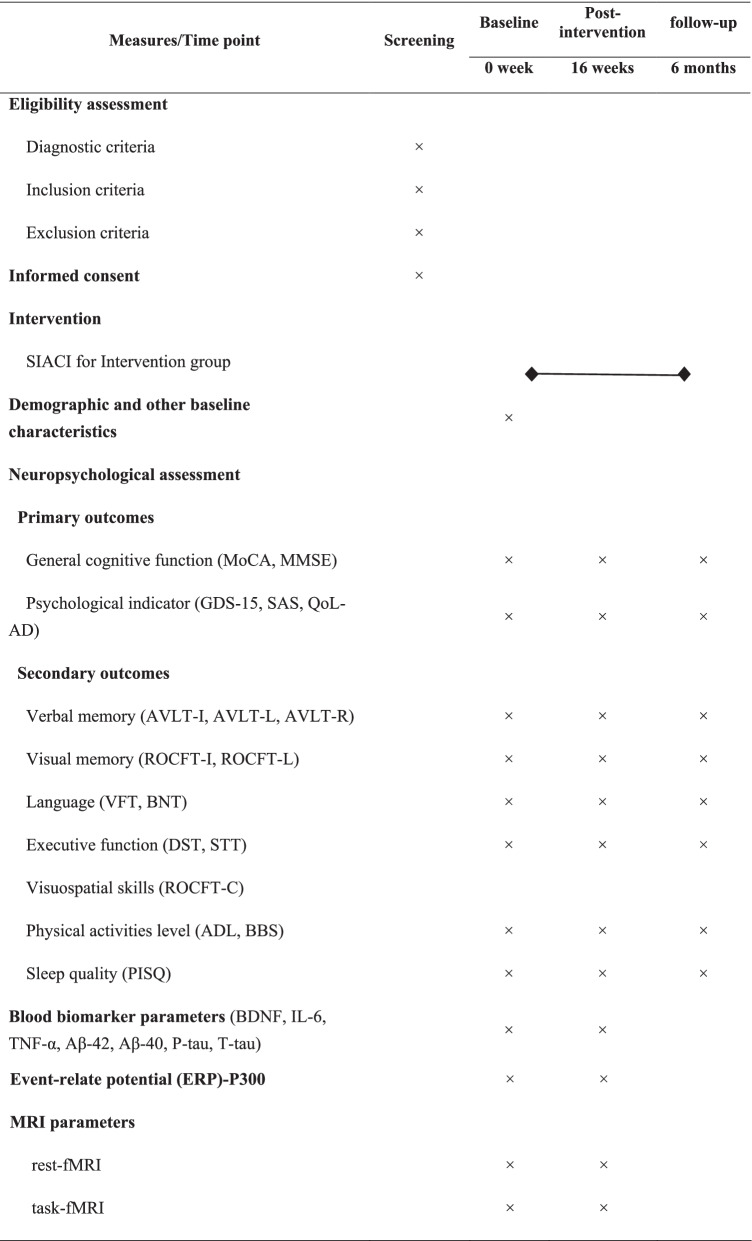
SIACI outcome measures

#### Demographic and other baseline characteristics

To determine whether demographic and other baseline variables are confounding factors that affect the outcome variables, basic demographic data (e.g., age, gender, religion, marital status, education, occupation, and socioeconomic status), history of disease, medication use, and leisure activities will be collected from participants via a questionnaire at baseline.

#### Neuropsychological assessments

##### Primary outcome measures

The primary outcome measures will be general cognitive function and psychological indicators, as measured by the Chinese versions of MoCA, MMSE, Geriatric Depression Scale (GDS) [54], Zung Self-Rating Anxiety Scale (SAS) [55], and Quality of Life in Alzheimer’s Disease scale (QoL-AD) [56].

##### Secondary outcome measures

The secondary outcome measures will include several commonly used measures of specific domains of cognitive function, sleep quality, and physical activity level. The measures of specific domains of cognitive function (memory, language, executive function, and visuospatial skills) will comprise the Auditory Verbal Learning Test (AVLT) [57], Category Verbal Fluency Test (CVFT) [58], Boston Naming Test (BNT) [59], Shape Trail Test (STT) [60], Digital Span Test (DST) [61], and Rey-Osterrieth Complex Figure Test (ROCFT) [62]. The Pittsburgh Sleep Quality Index (PSQI) [63] will be used to measure sleep quality, and the Berg Balance Scale (BBS) [64] and ADL will be used to measure the physical activity level. More information about the sensitivity and specificity of the assessment methods etc. can be found from the reference cited.

#### Blood biomarkers parameters

Before and after the intervention (in the morning after a 12-h fast), 5 mL whole blood will be collected into a polypropylene tube containing ethylenediaminetetraacetic acid (EDTA) from each participant’s antecubital vein by a registered nurse. The samples will immediately be centrifuged at 4000 rpm for 5 min at 23 °C (5810R Centrifuge, Eppendorf). The plasma will be aliquoted and stored at − 20 °C. Thereafter, the plasma levels of BDNF, IL-6, TNF-α, Aβ, and phosphorylated tau will be measured by enzyme-linked immunosorbent assays. The procedures will use the same types of assay kit and laboratory instruments, and they will be performed by the same laboratory technician to avoid inter-operator bias.

#### Event-relate potential P300

A Natus Dantec™ Keypoint Focus 8-channel system (Natus Medical Incorporated) will be used to record and analyze brainstem auditory ERP-P300 data. Each participant will sit in a comfortable chair in a sound-attenuated room with a warm light. Based on the international 10–20 system of electrode placement, central (Cz), parietal (Pz), and frontal (Fz) electrodes will be used for continuous recording; the right mastoid will serve as the reference electrode site. The auditory oddball paradigm, which consists of a series of standard (1000 Hz) and deviant (2000 Hz) tones, will be delivered binaurally through a headset. The sound level for each tone will be 110 dB, with a bandwidth of 0.5–20 Hz, a duration of 50 ms, and an inter-stimulus interval of 800 ms on average. All recordings will be performed with electrode impedances < 5 kΩ. The deviant tones will make up 20% of the tones and each of the three rounds of testing average count 20 stimuli. Each participant will be instructed to close their eyes, keep awake and concentrate, and press the button as accurately and quickly as possible when they hear the deviant sounds. Each participant will perform a pre-test first to make sure that they have understood the task. P300 latency (mV) and amplitude (ms) are key factors used for analyzing ERP data. Also, the reaction time (ms) is defined as the length between hearing a deviant tone and pressing the button.

#### MRI parameters

Multimodal MRI scans will be acquired at baseline and immediately after the intervention using a 3.0 T Prisma scanner (Siemens, Erlangen, Germany) in the same scan modes, which will involve structural MRI (sMRI), resting-state fMRI (rs-fMRI) and task-state fMRI (ts-fMRI). High-resolution T1-weighted images of the whole brain will be obtained using a sagittal 3D magnetization-prepared rapid gradient echo (MP-RAGE) sequence. Rs-fMRI will be performed using a multiband echo-planar imaging sequence. Ts-fMRI will comprise a scan involving memory and executive tasks. The scan sequence details are displayed in the Additional file. Quality-control checks on the phantom and imaging sequences will be performed before data collection. Image quality will be validated by an experienced imaging specialist. Individuals with potential cerebral impairments or structural abnormalities revealed by MRI will be excluded from subsequent experiments (e.g., rs-fMRI scan and ts-fMRI scan).

### Data collection and analysis

All data analysis will be performed using SPSS Statistics v22.0 (IBM). To ensure that the outcome assessors and data analysts are blinded to group assignment, they will not interact with the participants except, in the case of outcome assessors, during data collection. Demographic and other baseline characteristics will be summarized using descriptive statistics. If necessary, the results will be adjusted for potential confounders, such as age, gender, and education. Between- and within-subject comparisons of the neuropsychological test scores and other outcomes will be conducted using repeated-measures analysis of variance (ANOVA). Statistical analyses will be based on an intention-to-treat (ITT) approach [65], missing data will be imputed by multiple imputation. *P* < 0.05 for two-tailed tests will be considered significant. In sensitivity analyses, we will perform per-protocol (PP) analyses [66], including only the participants who completed the SIACI program and outcome assessment. The details of the fMRI data analysis are displayed in the Additional file.

## Discussion

The aim of the trial is to assess the effectiveness and mechanisms of a novel and innovative SIACI program for older adults facing cognitive decline. Although no curative pharmacotherapy currently exists for AD, a growing number of studies have documented the significant benefits of AT-based activities. These activities can increase participants’ motivation to engage in targeted cognitive and combined training programs, thereby improving their outcomes, such as communication, attention, pleasure, and neuropsychiatric symptoms of dementia and other CIs [15, 16, 26, 46, 67]. Art can play a key role in facilitating lifelong learning, in terms of discovering and building new skills and making meaning of experiences, because through art one’s past and present thoughts can be combined. AT, with appropriate modifications, has been shown to be suitable for individuals in the earliest stages of SCD to the later stages of severe CIs [27]. According to current evidence, no AT type is significantly more effective in individuals with dementia than the other types [28]. Additionally, the meta-analysis found that studies on a combination of visual art, music, and drama interventions found significant improvements in at least one outcome measure, such as mental stimulation, verbalization, personal control, positive emotional reactions, satisfaction, and self-esteem [28]. This suggests that a combination of AT types that engage participants in a variety of art activities may be effective for combating cognitive decline [28].

Art activities can improve the functions of neural networks related to various brain regions involved in emotions, self-control, cognition, and behavior. They do this by training brain regions to promote cooperation among these regions, stimulating the hypothalamus–pituitary–adrenal axis, regulating the excitement or inhibition of sympathetic system, and promoting the release of active substances known to be related to health (such as norepinephrine and acetylcholine) and thereby increasing BDNF [33, 68, 69]. In addition, cytokines play a central role in neuro-immune-endocrine processes, and the potent inflammatory cytokines IL-6 and TNF-α (which are products of activated microglia and astrocytes and are up-regulated in AD brains) are known to influence cognition via diverse mechanisms. High TNF-α levels during aging and in AD may contribute to amyloidosis [34, 70, 71]. As circulating peripheral levels of neuroprotective growth factors and inflammatory cytokines have been suggested to be associated with AD-type brain pathology (e.g., decreased Aβ-42 and increased phosphorylated tau) [37, 72, 73], identifying a means of modulating the levels of neuroprotective growth factors and pro-inflammatory cytokines would represent an important advance in AD treatment [74]. Moreover, the brain structures and functions involved in artistic processes (including how the brain operates during non-goal-directed compared to goal-directed behavior and creative thinking) have been investigated, the therapeutic factors involved above in AT have been hypothesized based on this, and neuroscience has thus been involved in designing and implementing effective interventions [75]. Experiences are crucial in bringing about neuroplastic change, and AT can offer direct and indirect experiences, which may underlie changes in the involvement of different brain structures and functions. However, there is a lack of evidence on the beneficial effects of art-based cognitive interventions on cognitive function, especially regarding neuroprotective and neuroinflammatory biomarkers and neurocognitive performance.

To the best of our knowledge, our trial is the first study to assess the effects of an art-based cognitive intervention in different stages of AD and to try to reveal the underlying blood biological and neural mechanisms from a scientific perspective. The trial will employ rigorous methods to reduce bias, such as randomization, proactive recruitment strategies, stringent eligibility screening, innovative theory-based intervention design, blinding of the outcome assessors and data analysts, and statistical analysis according to the ITT principle.

### Limitations

A limitation of the trial is that the participants and intervention staff cannot be blinded because it is difficult to conduct blinding in non-pharmacological trials. Also, bias in participant selection will be unavoidable. Moreover, the uncertainty regarding COVID-19 will bring challenges to the trial.

## Conclusion

In conclusion, the trial will elucidate the immediate and long-term effects of SIACI, involving neuropsychological testing and blood biomarkers. Neuroscience involving ERP-P300 and MRI parameters will make it possible to explore the mechanisms of SIACI in older adults with CIs, allowing the relationship between subjective and objective indicators to be investigated. These results will provide evidence on the effectiveness of the art-based cognitive intervention, which may delay or even halt cognitive decline in preclinical AD and AD itself.

## Supplementary Information

**Additional file 1.** Examples of Recruitment Poster. MRI data collection and analysis.

## Data Availability

The datasets generated during and/or analyzed during the present study will be available from the corresponding author on reasonable request.

## Abbreviations

AD: Alzheimer’s disease
ADL: Activities of daily living
ANOVA: Repeated-measures analysis of variance
AT: Art therapy
AVLT: Auditory verbal learning test
BBS: Berg balance scale
BDNF: Brain-derived neurotrophic factor
BNT: Boston naming test
CIs: Cognitive impairments
CVFT: Category verbal fluency test
DST: Digital span test
ERP: Event-related potential
ETC: Expressive therapy continuum
GDS: Geriatric depression scale
IL-6: Interleukin-6
ITT: Intention to treat
TNF-α: Proinflammatory cytokine tumor necrosis factor-α
NINCDS-ADRDA: Neurological and communicative disorders and stroke-Alzheimer disease and related disorders association
MCI: Mild cognitive impairment
MDV: Media dimension variables
MMSE: Mini-mental state examination
MoCA: Montreal cognitive assessment
MP-RAGE: Magnetisation- prepared rapid gradient echo
MRI: Magnetic resonance imaging
PP: Per-protocol
PSQI: Pittsburgh sleep quality index
QoL: Quality of life in Alzheimer’s disease scale
ROCFT: Rey-osterrieth complex figure test
rs-fMRI: Rest-state fMRI
SAS: Zung self-rating anxiety scale
SCD: Subjective cognitive decline
SCD-I: Subjective cognitive decline initiative
SIACI: Staged integral art-based cognitive intervention
sMRI: Structure MRI
STT: Shape trail test
ts-fMRI: Task-state fMRI
