# Correction: Effects of a staged integral art-based cognitive intervention (SIACI) program in older adults with cognitive impairments: protocol for a randomized controlled trial

**DOI:** 10.1186/s12877-022-03109-0

**Published:** 2022-05-17

**Authors:** Yuan-jiao Yan, Ming-ping Ma, Wen-chao Cai, Chen-shan Huang, Rong Lin, Yu-fei Chen, Hong Li

**Affiliations:** 1 Research Center for Nursing Theory and Practice, Fujian Provincial Hospital, Shengli Clinical Medical College of Fujian Medical University, No. 134 Dongjie Street, Gulou district, Fuzhou, 350001 Fujian Province China; 2 Department of Nursing, Fujian Provincial Hospital, Shengli Clinical Medical College of Fujian Medical University, No.134 Dongjie Street, Gulou district, Fuzhou, 350001 Fujian Province China; 3 The School of Nursing, Fujian Medical University, No.88 Jiaotong Road, Fuzhou, 350004 Fujian Province China; 4 Department of Radiology, Fujian Provincial Hospital, Shengli Clinical Medical College of Fujian Medical University, No. 134 Dongjie Street, Gulou district, Fuzhou, 350001 Fujian Province China; 5 Department of Chinese Language and Literature, Xiamen University, No. 422 Siming South Road, Xiamen, 361005 Fujian Province China

**Correction to: BMC Geriatr 22, 296 (2022)**

**https://doi.org/10.1186/s12877-022-02961-4**

After publication of this article [1], the authors reported that the details for affiliation 1 were incorrectly given as ‘Research Center for Nursing Theory and Practice, Fujian Provincial Hospital, No. 134 Dongjie Street, Gulou District, Fuzhou 350001, Fujian Province, China’ but should have been ‘Research Center for Nursing Theory and Practice, Fujian Provincial Hospital, Shengli Clinical Medical College of Fujian Medical University, No. 134 Dongjie Street, Gulou district, Fuzhou 350001, Fujian Province, China’.

The original article [1] has been updated.
