# Supplementary material for: Effects of a staged integral art-based cognitive intervention (SIACI) program in older adults with cognitive impairments: protocol for a randomized controlled trial
Source: BMC Geriatr. 2022 Apr 7;22:296. doi: 10.1186/s12877-022-02961-4 (PMC8988539; doi:10.1186/s12877-022-02961-4)
Supplement: Supplementary file 1 — Additional file 1. Examples of Recruitment Poster. MRI data collection and analysis. [file 12877_2022_2961_MOESM1_ESM.docx]

**Additional file**

**Effects of a staged integral art-based cognitive intervention (SIACI) program in older adults with cognitive impairments: protocol for a randomized controlled trial**

**Key Words:** Art; Non-pharmacological intervention; Older adults; Randomized controlled trial

**Running Head:** Effects of SIACI in Older Adults with CIs

**List of additional files**

**1. Examples of Recruitment Poster**

**2. MRI data collection and analysis**

**3. Reference**

1. **Examples of Recruitment Poster**


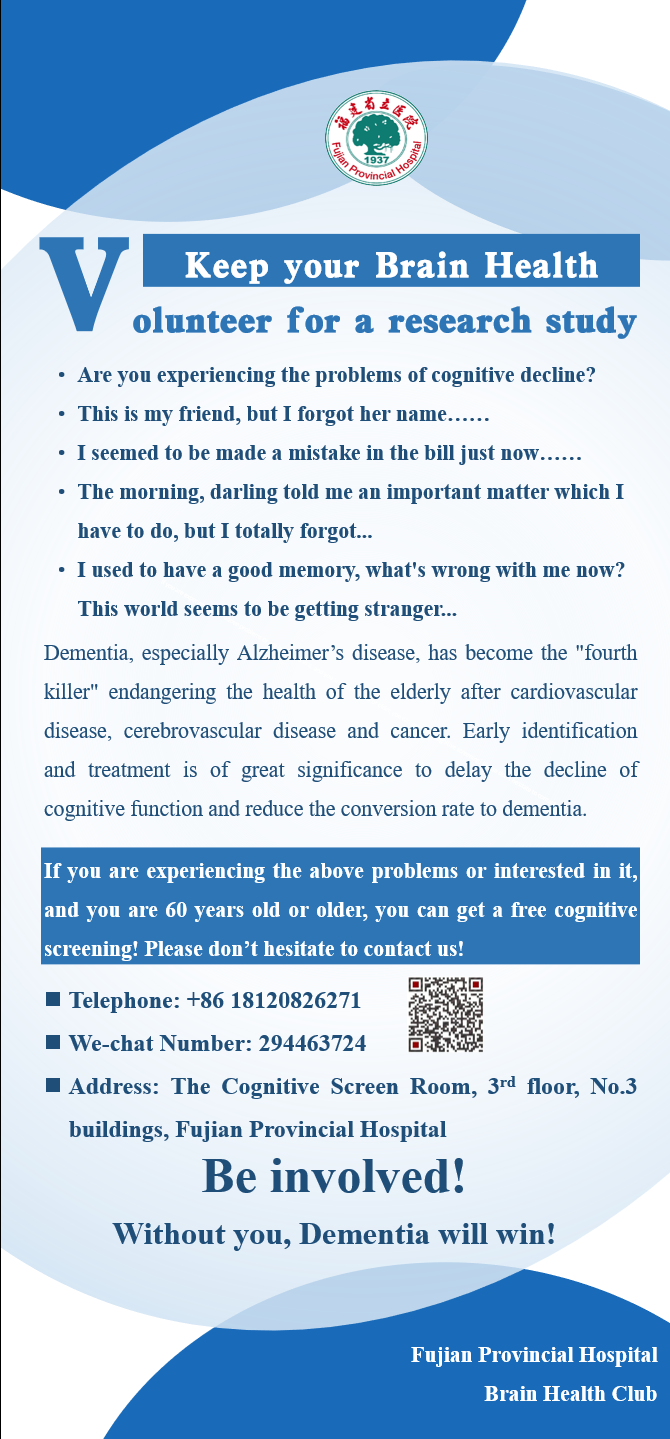


**Figure 1.** Details of the volunteer recruitment poster.


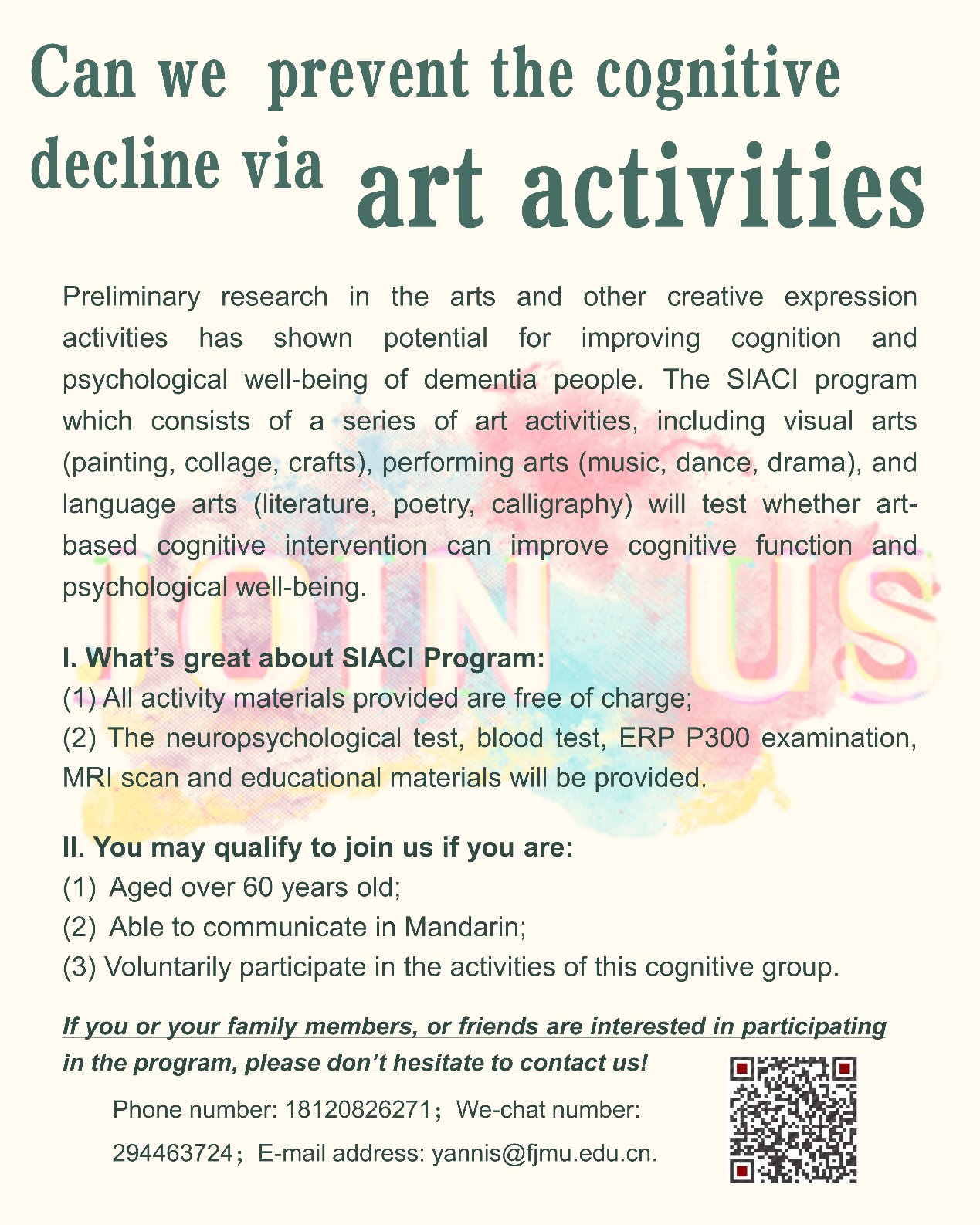


**Figure 2.** Details of the SIACI program recruitment poster.

**2.MRI data collection and analysis**

**2.1 MRI data collection**

The sMRI scan will include T1- and T2-weighted and T2-FLAIR imaging, and the time needed for the MRI protocol is estimated to be 35-50 minutes. High-resolution T1-weighted images of the whole brain will be obtained using a sagittal 3D magnetization prepared rapid gradient echo (MP-RAGE) sequence with the following parameters: repetition time (TR)=2530ms; echo time (TE)=2.98ms; time of inversion (TI)=1100ms; slice thickness=1 mm; 192 slices; flip angle=7°; voxel size=1.0×1.0×1.0 mm^3^; field of view (FOV)=256×256 mm^2^. The T2-weighted imaging will be obtained using a blade turbo spin echo (TSE) sequence with the following parameters: repetition time (TR)=3800ms; echo time (TE)=75ms; field of view (FOV)=220 × 220 mm^2^; 20 slices; flip angle=150°; voxel size=0.7×0.7×5.0 mm^3^; and slice thickness/gap= 5 mm/1.5mm. The T2-FLAIR imaging will be obtained using a turbo spin echo (TSE) sequence with the following parameters: repetition time (TR)=7500ms; echo time (TE)=83ms; time of inversion (TI)=2298ms; field of view (FOV)=220 × 220 mm^2^; 20 slices; flip angle=150°; voxel size=0.7×0.7×5.0 mm^3^; slice thickness/gap= 5 mm/1.5mm. Diffusion-weighted imaging is also used to exclude acute infarction with the following parameters: repetition time (TR)=2730ms; b-value=0/1000s/mm^2^ with echo time (TE)=58/98ms; field of view (FOV)=220 × 220 mm^2^; 20 slices; flip angle=180°; slice thickness/gap= 5 mm/1.5mm.

Rs-fMRI will be conducted using a multiband echo- planar imaging sequence with the following parameters: TR=2000ms; TE=30ms; 33 slices; voxel size=3.5×3.5×3.5 mm^3^; flip angle=90°; FOV=224×224 mm^2^; measurements=240; and time will last 8min6s.

For ts-fMRI, a scan involving memory and executive tasks will be conducted to explore the potential neural mechanisms of the intervention. Response memory and executive function before and after the intervention will be investigated using the classic visuospatial n-back task and Go/NoGo task, respectively. The tasks will involve the use of E-Prime 3.0 software (Psychology Software Tools Inc., Pittsburgh, PA, USA), and the stimuli will be presented using the Visual & Audio Stimulation System for fMRI (SA-9900; Shenzhen Sinorad Medical Electronics Co., Ltd., China), which will record the participants’ key press responses. This system is a professional stimulation system for fMRI, with a high-precision, high-brightness back-projection MRI-compatible projection system, a participant response feedback system, a synchronization system, and a high-quality, noise-reducing dual-way non-magnetic voice system, which can synchronize the presentation and scanning.

The procedure and parameters of the visuospatial n-back task (with 0-, 1-, and 2-back conditions) will be as reported in Ragland et al and Dong et al [1,2]. A target:foil ratio of 1:2 (i.e., 33% targets) will be maintained throughout. Each block will involve a stimulus duration of 1,000 ms and an interstimulus interval of 2,000 ms. There will be 15 blocks presented for each condition, and each condition will be repeated three times in pseudorandom order, giving a total of 135 stimuli. There will be a 9-s delay at the start of the task and between conditions, during which an instruction screen will appear informing the participant of the upcoming condition. This delay will also allow the participant to rest, and it will allow recovery of the hemodynamic response from the previous condition. The total task time will be 500 s. An illustration of the task is provided in Figure 3.

The procedure and parameters of the Go/NoGo task will be as reported in Menon et al [3]. In the Go and Go/NoGo blocks, each presentation of letters will be synchronized with the beginning of the scan. During the rest condition, participants will passively view a “+” on a blank screen. There will be two experimental conditions: Block A (the Go condition) will have 13 targets and Block B (the Go/NoGo condition) will have 7 targets and 5 non-targets. During the experiment, participants will view a series of letters once every 2s (1,000-ms stimulus and 1,000-ms interstimulus interval) and respond with a key press to every letter except the letter “V”. Each participant needs to respond using the forefinger of the right hand. In the Go (control) condition, participants will be presented a random sequence of letters other than the letter “V”. In the Go/NoGo (experimental) condition, participants will be presented with the letter “V” 50% of the time, thus requiring response to half the trials (Go trials) and response inhibition to the other half (NoGo trials). Each participant will perform four blocks of 336 s, each including 156 trials. Prior to their scan, each participant will be given instructions along with a practice test. Only participants with an accuracy rate of >80% will be included in the analyses.


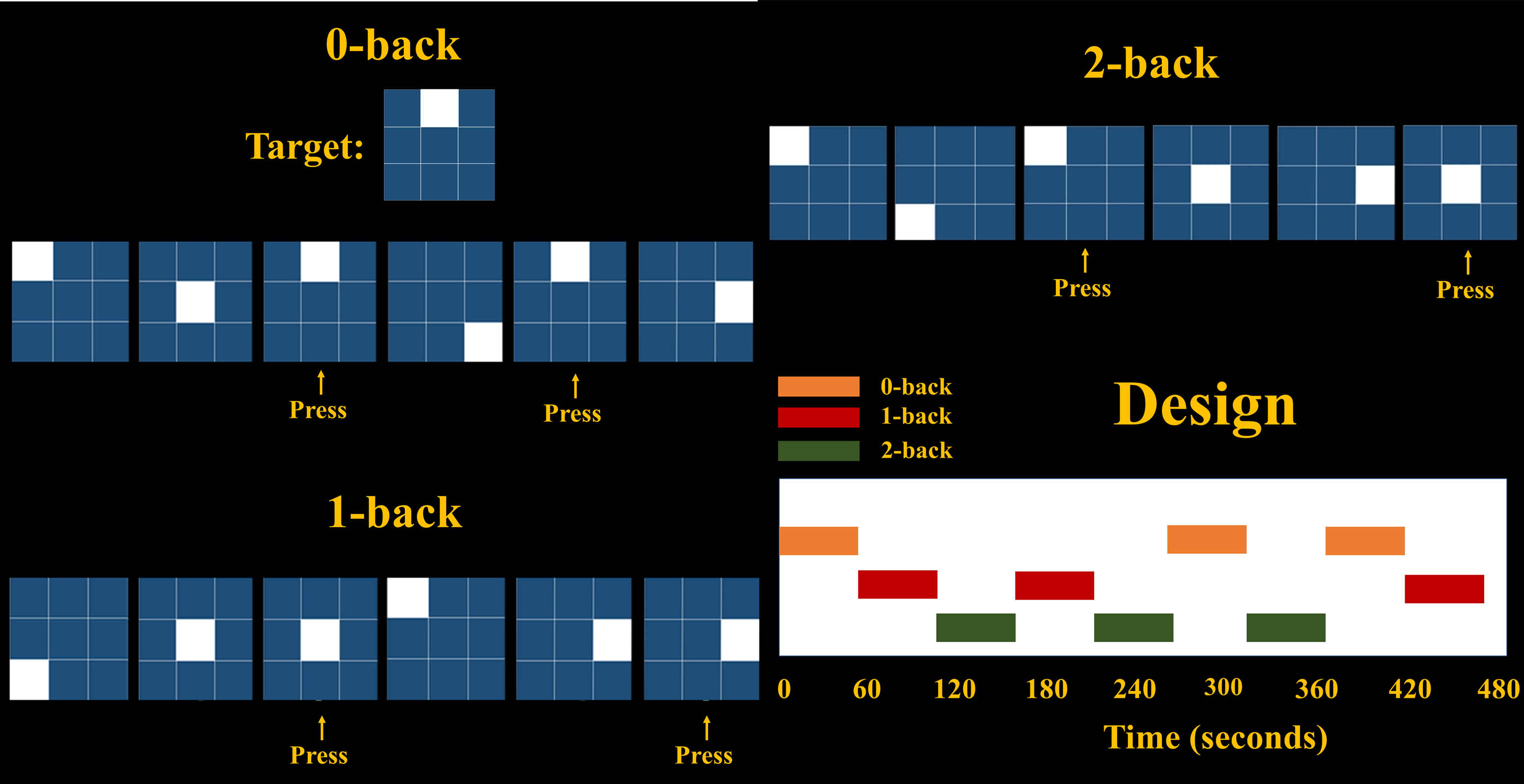


**Figure 3.** Illustration of the visuospatial n-back task stimuli and the blocked functional magnetic resonance imaging design.

**2.2 MRI data analysis**

Functional and task behavioral data will undergo a quality control evaluation and initial pre-processing using FBIRN tools and dashboard monitoring (www.birncommunity.org). Quality control ensure adequate data quality and consistency throughout the study. Errant subjects will be flagged for removal for either of two reasons: 1) average task performance at or below chance levels (<60 % accuracy), or 2) the concurrence of excessive motion (Signal-To-Noise-Fluctuation<65) and null functional activation in occipital lobes during visual stimuli [4].

fMRI data will be pre-processed using SPM v12 (https://www.fil.ion.ucl.ac.uk/spm/software/spm12/) in MATLAB 2013b (MathWorks, Natick, MA, USA), which will consist of slice-timing correction, realignment, co-registration, segmentation, spatial normalization, and Gaussian spatial smoothing; the signal from white matter and cerebrospinal fluid and their first-order derivatives will be included as confounders. In both groups, the regions of interest (ROIs) corresponding to visual cortical and subcortical networks of both hemispheres will be determined. Whole-brain tractographic reconstructions will be performed on pre-processed diffusion images. Connectivity between areas will be determined for each participant. For each pathway, we will extract the following diffusion tensor parameters: fractional anisotropy (FA), mean diffusivity (MD), and linear (Cl), planar (Cp) and spherical (Cs) coefficients. Comparisons between the two time points and between groups will be conducted using general linear models.

**2.2.1 N-back data analysis**

This image-based analysis will be performed for three contrasts: 1-back minus 0-back, 2-back minus 0-back, and 2-back minus 1-back. Contrasts will be performed both within and between tasks. Within-task contrasts will be performed on the whole brain. Between-task comparisons for each contrast will be restricted to voxels that had above-threshold responses for either task during their respective within-task contrasts. This conservative approach will ensure that between-task contrasts will be limited to hypothesized regions showing reliable task-related activations.

Responses will be recorded as true positive (TP), false positive (FP), true negative, and false negative. Discriminability (Pr) will be calculated as a measure of performance success, and TP RT calculated as a measure of performance effort. Pr values and median RT will be entered into a repeated measures analysis of variance (ANOVA; Proc GLM, general linear procedure; SAS Institute, 1996) to examine main effects of load (0-back, 1-back, 2-back).

**2.2.2 Go/Nogo data analysis**

A General Linear Model for each of the four runs will be performed, predicting fMRI time series data with correct Go and NoGo trials, convolved with a double gamma hemodynamic response function, along with their temporal derivatives. The four runs will be then collapsed into visit level maps using fixed effects prior to calculating voxel wise group statistics mixed effects analysis with correction for multiple comparisons.

For group analysis, a random effects model will be used to determine voxel-wise t-statistics contrasting specific conditions of interest. This model estimates the error variance for each condition of interest across subjects, rather than across scans. The random effects model provides better generalization to the subject population, albeit with some loss in power due to averaging in the time domain. This analysis proceeded in two steps. In the first step, adjust images corresponding to the conditions/ events of interest were determined. For each condition, a weighted average of the images is computed taking into account the haemodynamic response. In the second step, these condition-specific images are contrasted in a general linear model to determine appropriate t-statistics. The t-statistics are normalized to Z scores to determine significant clusters of activation. The analysis consists of all successful trials.

For each subject, mean images corresponding to correct NoGo events (“correct NoGo”) and false alarm events (“incorrect NoGo”) will be computed for each subject. Brain activation during error processing will be estimated using an event-related contrast of “incorrect NoGo” and “correct NoGo” events. Missed Go events will not be used in calculations of error processing in order to avoid possible confounds resulting from different types of errors (i.e., errors of commission vs. errors of omission).

In addition, mean images will be derived for each of the three conditions: (1) Go (control), (2) Go/NoGo (experimental), and (3) rest. These images will be contrasted (Go/NoGo versus Go) to determine brain activation during response inhibition and competition using a blocked design in order to compare this type of neural process with that of error processing. Brain activation related to motor response execution is investigated using a (Go versus Rest) contrast. Neuroanatomical locations of activation will be presented in normalized MNI space.

**2.2.3 Behavioral data analysis**

The reaction time (RT) and number of correct responses and misses to Go events are computed separately for the Go and Go/NoGo condition. The number of correctly withheld responses to the NoGo events, and the number of false alarm (FA) responses and their RTs, are computed. Percent correct and incorrect responses and RTs are compared using students’ t-test.

**Reference**

1. Ragland JD, Turetsky BI, Gur RC, Gunning-Dixon F, Turner T, Schroeder L, et al. Working memory for complex figures: an fMRI comparison of letter and fractal n-back tasks. Neuropsychology. 2002;16:370–9.

2. Dong S, Wang C, Xie Y, Hu Y, Weng J, Chen F. The impact of abacus training on working memory and underlying neural correlates in young adults. Neuroscience. 2016;332:181–90.

3. Menon V, Adleman NE, White CD, Glover GH, Reiss AL. Error-related brain activation during a Go/NoGo response inhibition task. Hum Brain Mapp. 2001;12:131–43.

4. Glover GH, Mueller BA, Turner JA, van Erp TGM, Liu TT, Greve DN, et al. Function biomedical informatics research network recommendations for prospective multicenter functional MRI studies. J Magn Reson Imaging. 2012;36:39–54.
